# Correction: TFEB and MCOLN1 are important for *Coxiella burnetii* egress via lysosomal exocytosis

**DOI:** 10.3389/fcimb.2026.1974473

**Published:** 2026-09-07

**Authors:** Sven Rinkel, Jan Schulze-Luehrmann, Bhavyalakshmi Kadavil Baburaj, Fiona Weber, Elisabeth M. Liebler-Tenorio, Anja Lührmann

**Affiliations:** 1 Mikrobiologisches Institut, Universitätsklinikum Erlangen, Friedrich-Alexander-Universität Erlangen-Nürnberg, Erlangen, Germany; 2 Friedrich-Loeffler-Institut, Institut für Molekulare Pathogenese, Jena, Germany

**Keywords:** *Coxiella burnetii*, Egress, LAMP, lysosomal exocytosis, MCOLN1, Q fever, TFEB, type IV secretion system

There was a mistake in **Figure 7** as published. Part of the figure (**Figures 7D–F**) was removed and instead part of **Figure 8** (**Figures 8A, B**) was included. The corrected **Figure 7** appears below.

**Figure 7:**
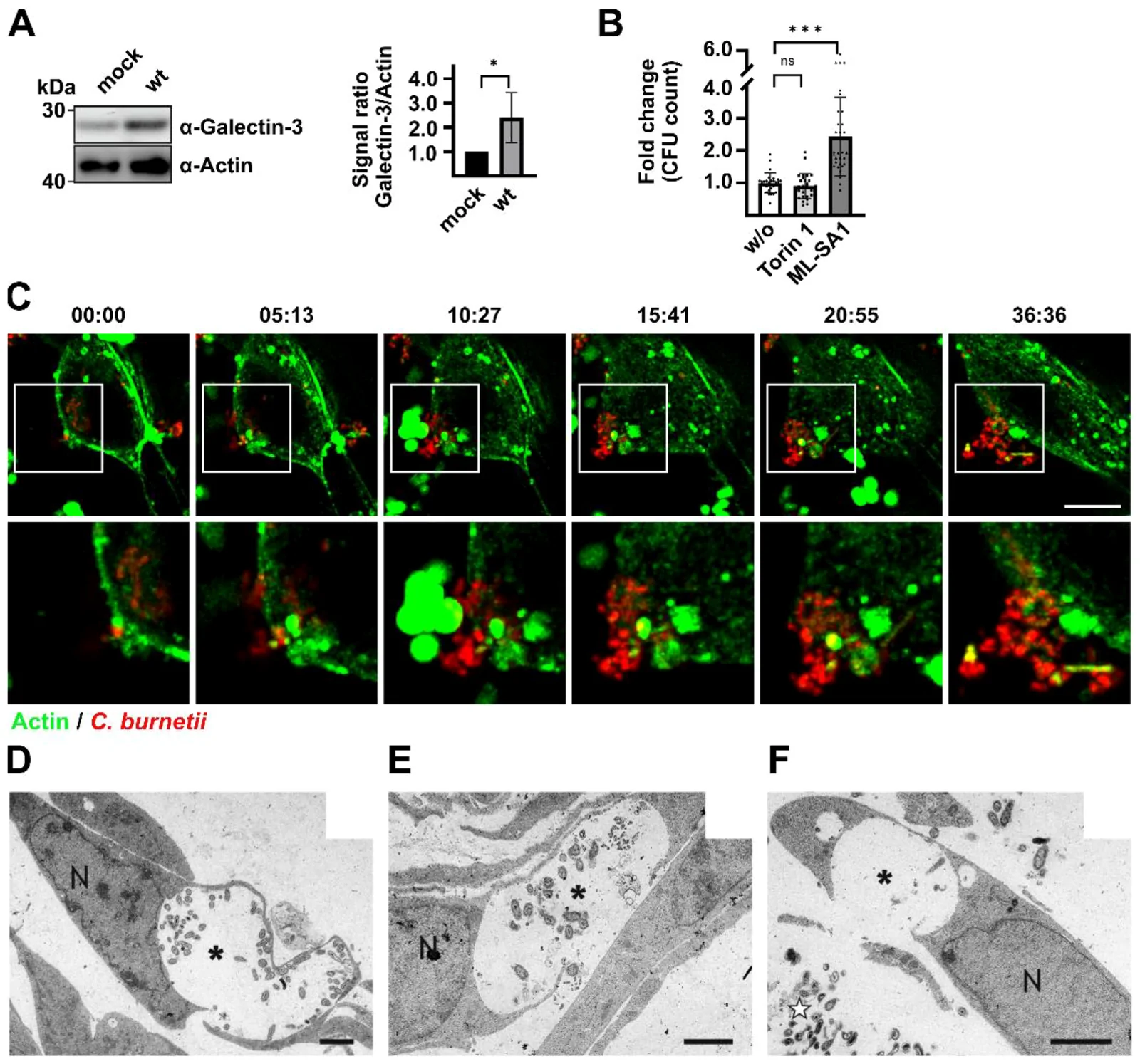
*C. burnetii* egresses via lysosomal exocytosis. **(A)** HeLa cells were either not infected (mock) or infected with wild-type *C. burnetii* (wt) at MOI 200. Cell lysates were analyzed by Western blot using antibodies against Galectin-3 and Actin, the latter serving as loading control. Representative immunoblots out of three independent experiments with similar results are depicted. Densitometric analysis of the Galectin-3/Actin ratio was performed using ImageJ. Arbitrary units (AU) are shown relative to uninfected cells (mock). Mean ± SD, n=3, one sample t-test and unpaired Student’s t-test. *p < 0.05. **(B)** HeLa cells were infected with wild-type *C. burnetii* at MOI 200 for 6 h. Cells were washed ten times and were treated without (w/o), with Torin 1 (250 nM) or with ML-SA1 (25 µM) for 18 hours. The CFU counts of the supernatants from three independent experiments performed in triplicates are shown. The fold change in CFU relative to untreated cells (w/o) are shown. The bar represents the mean. The Mann-Whitney test was performed with GraphPad Prism. ns=not significant, ***p < 0.001. **(C)** EA.hy926 cells were infected with *C. burnetii* at MOI of 200. After 24 h of infection, the cells were washed five times with PBS and treated with gentamicin (200 µg/ml) for 4 h to eliminate extracellular bacteria. Five days post-infection, the cells were visualized by spinning disc microscopy. The actin cytoskeleton was stained with SiR-actin (green). Images were acquired every ~5 min. Time stamp: min:sec. Scale bar: 10 µm. The white inlet shows a 4-fold magnification. **(D–F)** Wild-type MEFs were infected for 7 days with *C. burnetii*. The cells were fixed with glutaraldehyde, embedded in araldite and ultrathin sectioned for transmission electron microscopy. Open CCVs (marked with *) release *C. burnetii* into the extracellular space (marked with white stars). All cells affected have normal nuclear (marked with N) morphology and no signs of cellular damage. The opening of the CCV in **(D)** is small, larger in **(E)** and extensive in **(F)**. While in **(D, E)**, *C. burnetii* are still found in the CCVs, the CCV in **(F)** is empty, with numerous bacteria in the extracellular space. Scale bar: 2 µm.

The original version of this article has been updated.

