# Supplementary material for: TFEB and MCOLN1 are important for Coxiella burnetii egress via lysosomal exocytosis
Source: Front Cell Infect Microbiol. 2026 Aug 14;16:1835140. doi: 10.3389/fcimb.2026.1835140 (PMC13521786; doi:10.3389/fcimb.2026.1835140)
Supplement: Supplementary file 1 [file SupplementaryFile1.docx]

**Supplementary information**

**Figure S1**

**Figure S1. Treatment with Torin 1 leads to a dose-dependent nuclear localization of TFEB.** HeLa cells were either not treated or treated with increasing concentrations of Torin for 24 hours. The cells were fixed, permeabilized and stained with an anti-TFEB antibody (green) and with DAPI (blue) to stain the nucleus. Representative immunofluorescence images are shown. Scale bar = 10 µm.

**Figure S2**

**Figure S2. Only infection with wild-type *C. burnetii* triggered nuclear migration of TFEB.** HeLa cells were either not infected (mock) or infected with *C. burnetii* wild-type (wt), the T4BSS mutant Δ*dotA* or the Δ*ankG* mutant at MOI 200 for 48 hours. Immunoblot analysis of GAPDH (marker for cytoplasmic fraction), Histone H3 (marker for nuclear fraction) and TFEB in the cytoplasmic and nuclear fractions are shown. Representative immunoblots of the cellular fractionation assay from one of two independent experiments with similar results are depicted.

**Figure S3**

**Figure S3. Ectopically expressed AnkG does not activate TFEB.** HeLa cells were stably transfected with doxycycline inducible AnkG. (A) Cells were either not treated (w/o), treated with Doxycycline to induce AnkG expression, treated with Torin 1 to activate TFEB or infected with wt *C. burnetii* at MOI 100 for 72 hours. The cells were fixed, permeabilized and stained with anti-TFEB and with DAPI. Representative immunofluorescence images are shown. Scale bar = 10 µm. Arrows indicate nuclear localization of TFEB. (B) Cell lysates were analyzed by Western blot using antibodies against AnkG and Actin as loading control. Representative immunoblots out of three independent experiments with similar results are shown.

**Figure S4**

**Figure S4. Galectin-3 localizes to the CCV.** HeLa cells were infected with wild-type *C. burnetii* (wt) at MOI 200 for 72 h. Cells were fixed, permeabilized and labeled with anti-Galectin-3 (green), anti-LAMP1/2 (red) and DAPI (blue). Representative immunofluorescence images are shown. Scale bar = 10 µm.

**Figure S5**

**Figure S5. ML-SA1- and Torin-treatment did not influence intracellular *C. burnetii* levels.** HeLa cells were infected with wild-type *C. burnetii* at MOI 200 for 6 h. Cells were washed ten times and were treated without (w/o), with Torin 1 (250 nM) or with ML-SA1 (25 µM) for 18 hours. *C. burnetii* were isolated and a CFU assay was performed. Three independent experiments were performed in triplicates. The data are shown as individual data points (mean ± SD). Significance was determined by a Mann-Whitney U test.

**Figure S6**

**Figure S6. CCVs disintegration at later stages of infection.** EA.hy926 cells were infected with *C. burnetii* expressing IPTG-inducible TagRFP fluorescent protein at MOI 200 for 5 days. Live cell images of infected cells in 35 mm glass bottom culture dishes acquired with an ApoTome. A representative image is shown. pCCV = peripheral CCV, cCCV = central/juxtanuclear CCV.

**Figure S7**

**Figure S7. Infected cells maintain their membrane integrity.** EA.hy926 cells, wild-type (wt) MEFs and LAMP1/2 knock-out (ko) MEFs were infected with *C. burnetii* at MOI 200 for 5 days. (left) The cells were stained with anti-LAMP1/2 and anti-*C. burnetii* and the percentage of infected cells were determined using confocal microscopy. In one hundred cells the presence of CCVs were scored in each of three independent experiments. Error bars indicate ± SD. (right) The percentage of cells with an intact membrane was evaluated by FDA (live stain) and PI (death stain) staining using FACS analysis. 30.000 event in each of three independent experiments were analyzed. Error bars indicate ± SD.
